# Subacute Toxicity Effects of the Aqueous Shoot Extract of *Yushania alpina* (K. Schum.) W.C.Lin in Sprague Dawley Rats: An Appraisal of Its Safety in Ethnomedicinal Usage

**DOI:** 10.1155/2022/6283066

**Published:** 2022-08-25

**Authors:** Joseph Ngugi Wanjiru, Timothy Elias Maitho, James Mucunu Mbaria, Gervason Apiri Moriasi

**Affiliations:** ^1^Department of Public Health, Pharmacology, and Toxicology, College of Veterinary and Agricultural Sciences, University of Nairobi, P. O. Box 29053-00625, Nairobi, Kenya; ^2^Department of Biochemistry, Microbiology and Biotechnology, Kenyatta University, P. O. Box 43844-00100-GPO, Nairobi, Kenya; ^3^Department of Medical Biochemistry, College of Health Sciences, School of Medicine, Mount Kenya University, P. O. 342-01000, Thika, Kenya

## Abstract

Plant-based medicines have effectively managed several ailments in humans and animals since prehistoric times. However, the pharmacologic efficacy and safety of many plants currently used in traditional medicine have not been explored empirically, which raises serious public health concerns, derailing further research and their integration into the conventional healthcare system. Despite the longstanding ethnomedicinal usage of *Yushania alpina* shoot extract to treat inflammation, microbial infections, and diarrhoea, among other diseases, there is insufficient scientific data to appraise its toxicity profile and safety. Accordingly, we investigated the subacute toxicity of the aqueous shoot extract of *Y. alpina* in Sprague Dawley rats (both sexes) for 28 days based on the Organisation for Economic Cooperation and Development guideline 407. In this study, all the experimental rats treated orally with 40 mg/Kg BW, 200 mg/Kg BW, and 1000 mg/Kg BW of the aqueous shoot extract of *Y. alpina* remained normal, like the control group rats, and did not show any clinical signs of subacute toxicity, and no morbidity or mortality was recorded. Besides, the weekly body weight gains and the haematological and biochemical parameters of experimental rats orally administered with the studied plant extract at the tested doses and in the control group were comparable (*P* > 0.05). No pathologic alterations in internal organs were observed following necroscopy. Further, the differences in weights of the liver, kidney, and spleen of experimental rats which were subacutely treated with the studied plant extract and the control rats were insignificant (*P* > 0.05). Moreover, no histopathological changes were observed in tissue sections of the liver, kidney, and spleen obtained from all the experimental rats. Our findings demonstrate that the aqueous shoot extract of *Y. alpina* may be safe as it does not elicit subacute toxicity in Sprague Dawley rats. Further toxicological and pharmacological studies using other model animals and in clinical setups are encouraged to fully appraise the efficacy and safety of the studied plant extract.

## 1. Introduction

Since antiquity, humans have practised herbalism, the major form of ethnomedicine, to manage various diseases [1]. Medicinal plants are relatively accessible, affordable, considerably efficacious, and safer than synthetic drugs, hence their prominence worldwide [2]. Indeed, the World Health Organization estimates that a higher proportion of the global population (80%) utilise herbals to meet their essential healthcare requirements, especially in less-developed countries such as Kenya [3, 4]. However, despite the enormous potential of medicinal plants in offering efficacious and safe lead compounds for drug development, due to the diverse array of pharmacologically active phytochemicals they produce, only a few have been investigated empirically [5–7].

The lack of standardised methods of preparation, formulation, storage, labelling, marketing, and clear dosage regimes of herbals for various diseases and patients and legislative frameworks governing traditional medicine have elicited safety and credibility concerns [8, 9]. In addition, there is insufficient empirical data on herb-herb and herb-conventional drug interaction and pharmacologic modes of action of various ethnomedicinally important plants, hampering the integration of herbal medicine into conventional healthcare [9–11]. Thus, it is imperative to empirically validate medicinal plants' pharmacologic efficacy and safety to validate their healing claims and valorise promising ones as alternative sources of therapies.

*Yushania alpina* (K. Schum.) W.C.Lin is the longest grass species of the Poaceae family, commonly known as “*Bamboo*,” which grows up to 40 metres high and is predominant in the temperate regions of Africa [12]. Ethnomedicinally, shoots of *Y. alpina* are used to treat ulcers, threadworms, diarrhoea, jaundice, cough, wounds, maggot infections, microbial infections, and inflammation, among other associated diseases [12–14]. Previous studies have shown that shoots of *Y. alpina* contain low-fat content and high amounts of vitamins (B6, Vitamin A, and Vitamin E), phosphorus, potassium, magnesium, carbohydrates, and dietary fibres, which are nutritionally essential to human health [12, 14, 15].

Li et al. [16] demonstrated the antiobesity effects of *Y. alpina* shoot fibre in experimental mice via the modulation of gut microbiota. Preliminary phytochemical screening has revealed the presence of glycosides and flavones among other bioactive amalgams of pharmacologic significance in shoot extract of *Y. alpina* [12]. Although *Y. alpina* has been used extensively in traditional medicine, there is scanty empirical information to validate its pharmacological potential, safety, and toxicity profile. Hence, this study was designed to explore the subacute toxicity of the aqueous shoot extracts of *Yushania alpina* to validate its healing claims in ethnomedicine and as an alternative source of efficacious and lead compounds for drug development.

## 2. Materials and Methods

### 2.1. Study Plant

The researchers collected fresh shoots of *Y. alpina,* assisted by a renowned herbalist, based on their ethnomedicinal usage in treating various diseases from the natural habitat in Kipipiri Subcounty in Nyandarua County (0° 33′ 0″ South and 36°′ 0″ East) in Kenya. Sample specimens were authenticated taxonomically at the National Museums of Kenya (NMK/BOT/CTX/1/2), and duplicates were deposited for future reference.

The samples were chopped and distributed on a wooden laboratory bench to dry naturally, away from direct sunlight, in a well-ventilated environment for three months. Occasional grabbling was done to deter moisture build-up and facilitate even drying. Afterwards, the material was ground using an electric plant mill, packaged in khaki envelopes, and stored on a laboratory shelf.

### 2.2. Extraction Procedure

The previously described hot maceration technique followed by *in vacuo* lyophilisation was adopted for aqueous extraction of the powdered plant material [17]. Briefly, the ground material (850 g) was soaked in three litres of distilled water in a conical flask and heated in a water bath set at 60^o^C for two hours. After that, the concoction was left to cool, double-filtered with cotton gauze and cotton wool plugs, and aliquoted (200 ml) into clean freeze-drying flasks. Then, the flasks were covered with dry carbon ice and acetone mixture, fitted into a freeze dryer equipment, and lyophilised *in vacuo* for 48 hours. The lyophilised extract was weighed, and percentage yield was determined, stored in a clean labelled, airtight sample bottle at 4^0^ C in a refrigerator, and retrieved when required.

### 2.3. Investigation of Subacute Toxicity

#### 2.3.1. Experimental Rats

Twenty male and twenty nulliparous and nonpregnant female rats weighing 180 ± 10 g and aged nine weeks were sourced from the breeding section of the Department of Public Health, Pharmacology, and Toxicology of the University of Nairobi. They were weighed and randomly assigned to respective experimental groups containing five rats, each according to the experimental schedule. They were marked on their tails using a permanent marker pen for easy identification and kept in polypropylene cages supplemented with softwood shavings under standard conditions (12-hour-light and 12-hour-dark cycle, 24 ± 1°C, and 55% humidity) in the research laboratory. The animals were supplied with rodent pellets and clean drinking water *ad libitum* and acclimatised for five days before experimentation. The animals were fasted overnight prior to dosing.

#### 2.3.2. Subacute Oral Toxicity Study

We adopted the subacute oral toxicity test guideline 407 described by the OECD to determine the toxicity of the plant extract in Sprague Dawley rats [18]. Briefly, the administration doses of the study extracts (40 mg/Kg BW, 200 mg/Kg BW, and 1000 mg/Kg BW) were prepared according to guidelines recommended by the OECD and administered by oral gavage into the study animals once daily for 28 days. The control group rats were orally administered with 1 ml/100 g BW of distilled water in the same manner as the extract-treated rats. All the animals were observed daily for any signs of toxicity, including feed and water intake, behavioural and neurological changes, abnormal secretions, and motor function changes, among other clinical symptoms of toxicity. The rats were also observed for mortality and were weighed on week 0 (day 0), week 1 (day 7), week 2 (day 14), week 3 (day 21), and week 4 (day 28), respectively. After the 28-day experimental period, the animals were observed for an extra day for delayed symptoms of toxicity.

#### 2.3.3. Blood Sampling and Analysis

Whole blood was collected from each experimental rat through the orbital sinus sampling technique into two vacutainers (a plain tube to separate serum for clinical biochemistry analyses and an ethylene diamine tetra-acetic acid (EDTA) tube for haematological analyses). An automated haematology analyser was used to determine the concentrations of various haematological parameters. Besides, the concentrations of various biochemical parameters in serum samples were measured using an automated biochemistry analyser.

#### 2.3.4. Necroscopy and Histopathology

Gross necropsies were performed according to standard procedures [18, 19] on all the experimental rats to observe any study extract-induced toxicity on the internal membranes, intestines, liver, heart, kidneys, lungs, and other internal organs. Then, the liver, kidneys, and spleen were harvested and fixed in 10% of neutral buffered formalin. The fixed samples were sliced, embedded in paraffin wax blocks, and processed histologically according to a standard procedure [20]. The processed samples were trimmed into 4 *μ*m thick sections, transferred into glass slides, and stained with routine haematoxylin and eosin stains. The stained sections were observed under a light microscope at magnifications of ×40 and ×100 (with oil immersion); any morphological changes and toxicological signs such as inflammation/ulceration, atrophy, cell infiltration, or deranged architecture, discolouration, and dead cells were recorded. Also, a digital camera attached to the light microscope captured photomicrographs of the observed sections, which were further reviewed and documented.

### 2.4. Data Management, Statistical Analysis, and Reporting

Qualitative data were recorded and presented in prose and in the form of photographs and micrographs. Quantitative data was entered into a spreadsheet (Microsoft 365) and transferred to Minitab version 21.1 software (State College Pennsylvania; Minitab, Inc. htpps://www.minitab.com) for analysis. The data were descriptively analysed, and the results were expressed as $\bar{x}\pm SEM$ of replicate measurements or observations. After that, inferential statistics were performed using a One-Way Analysis of Variance (ANOVA) to determine significant differences among means. After that, Tukey's *post hoc* test was used for pairwise comparison and separation of means. Besides, we performed an unpaired Student's *t*-test to compare the differences between two independent variables. Values with *P* < 0.05 were considered significant.

### 2.5. Ethical Considerations

This study was approved by the Faculty of Veterinary Medicine, Biosafety, Animal Use and Ethics Committee (BAUEC) of the University of Nairobi (FVM BAUEC/2020/261) and authorised by the National Commission for Science, Technology, and Innovation (NACOSTI) of Kenya (NACOSTI/P/21/11569). All the biological materials and samples were used and disposed of according to the guidelines stipulated by the BAUEC and NACOSTI.

## 3. Results

### 3.1. Wellness Parameters

In this study, all the experimental rats (both sexes) administered with the test extract appeared normal throughout the 28-day study period. Also, the treated rats did not exhibit any clinical, behavioural, motor, or neurological signs of extract-induced toxicity. No morbidity or mortality was observed in extract-treated rats, even at 1000 mg/Kg BW, during the 28-day experimentation period. Thus, the LD_50_ value of the studied plant extract was deemed to be >1000 mg/Kg·BW.

### 3.2. Feed and Water Intake

The results revealed no significant differences in the average daily feed intake in experimental rats (both sexes) treated with the test extract at all dose levels (*P* > 0.05; Table 1). Similarly, the average daily feed intake recorded in the male control group rats was comparable to those recorded in rats (male) administered 40 mg/Kg BW and 200 mg/Kg BW, respectively, of the test extract (*P* > 0.05; Table 1). Besides, the difference in average feed intake in female experimental rats treated with 40 mg/Kg BW of the study extract was significantly lower than that recorded for the female control group rats (*P* < 0.05; Table 1). Male rats' average daily feed intakes were significantly higher than those recorded for female rats (*P* < 0.05; Table 1).

The results showed no significant differences among the average daily water intakes recorded in male experimental rats (*P* > 0.05; Table 2). Generally, the average daily water intake by the male experimental rats was significantly higher than that of female rats (*P* < 0.05; Table 2). Besides, no significant differences in the average daily water intakes were observed between the female control rats and those (female) treated with 200 mg/Kg BW and 1000 mg/Kg BW of the extract and among those treated with the test extract at all doses (*P* > 0.05; Table 2).

### 3.3. Body Weight Gain

Weekly analysis of body weights of experimental rats (both sexes) treated orally with the aqueous shoot extract of *Y. alpina* and respective control group rats revealed no significant differences each week (*P* > 0.05; Table 3). Moreover, the average body weights of the experimental rats (both sexes) increased normally during the experimental period, with significantly higher body weights recorded in the fourth week compared with those recorded in week 0 (baseline) (*P* < 0.05; Table 3).

Notably, the differences in body weights of the control group rats (male and female) and those treated with 40 mg/Kg BW (male and female), 1000 mg/Kg BW (male), and 200 mg/Kg BW (female) of the test extract in the second week (week 2) and fourth week (week 4) were insignificant (*P* > 0.05; Table 3). Additionally, the differences in body weights recorded for all the experimental rats (male and female) between the first (week 1) and the second week (week 2) were insignificant.

### 3.4. Haematological Parameters

The WBC, RBC, Hb, MCHC, and HCT concentrations were comparable in all experimental rats (male and female) (*P* > 0.05; Table 4). Similarly, significant differences in PLT concentrations were observed among the control group rats (male and female), extract-treated female rats (all dose levels), and extract-treated male rats (40 mg/Kg BW and 200 mg/Kg BW) (*P* > 0.05; Table 4). Likewise, the differences in platelet levels recorded in rats (both sex) treated with 200 mg/Kg BW and 1000 mg/Kg BW of the extract and their respective control group rats (both sexes) were insignificant (*P* > 0.05; Table 4).

### 3.5. Biochemical Parameters

We determined the biochemical parameters of experimental rats treated with the test extract to assess its safety. The serum urea levels in experimental rats (both sexes) which received the test extract (at all dose levels) were comparable to those of the respective control group rats (*P* > 0.05; Table 5). Notably, extract-treated male rats had significantly higher concentrations of serum urea levels than those of female rats (control and extract-treated groups) (*P* < 0.05; Table 5).

The results further showed significantly higher serum creatinine concentrations in female rats orally administered with 40 mg/Kg BW of the test extract than those recorded in male rats treated with the same extract (200 mg/Kg BW) (*P* < 0.05; Table 5). The serum creatinine levels in the extract-treated rats and their respective controls were comparable (*P* > 0.05; Table 5).

Besides, the alkaline phosphatase levels in all the extract-treated experimental rats (both sexes), except the male rats, which received 1000 mg/Kg BW of the extract, were comparable (*P* > 0.05; Table 5). Notably, the male rats which received 1000 mg/Kg BW of the extract orally had significantly higher alkaline phosphatase levels than those recorded in all the female rats (*P* < 0.05; Table 5).

The results showed no significant differences in the alanine transferase, aspartate transaminase, and serum albumin levels among all the experimental rats (*P* > 0.05; Table 5). Likewise, the total protein levels recorded in male rats were comparable to those of female rats in this study, as shown in Table 5 (*P* > 0.05).

### 3.6. Gross Necroscopic Examination of Experimental Rats Treated with the Aqueous Shoot Extract of *Y. alpina*

**Figure 1:**
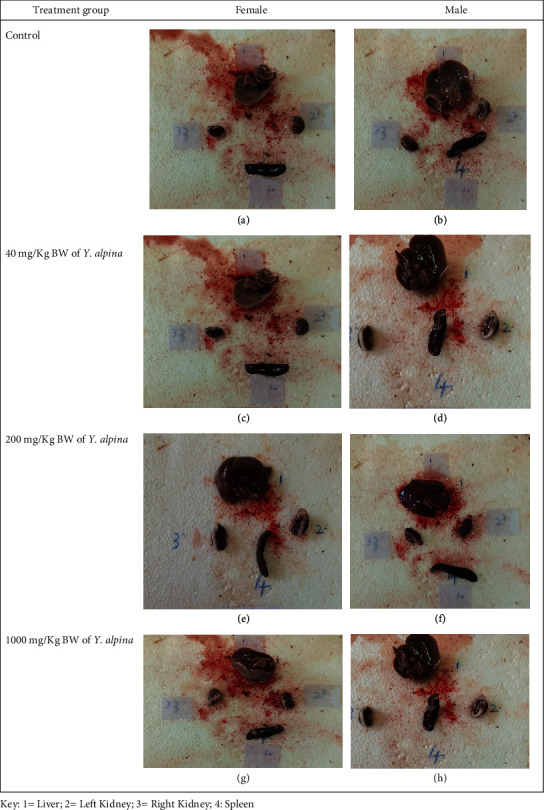
(a–h) Gross necroscopy of the liver, kidneys, and spleen of experimental rats.

Upon necroscopy, no gross alterations of appearance, colour, texture, haemorrhage, and size were observed in all extract-treated rats' internal organs (liver, spleen, kidney, heart, membranes, lungs, and intestines) (both sexes) compared to control group rats. Figures 1(a)–1(h) show representative gross necroscopy findings of this study.

### 3.7. Selected Organ Weights

This study observed insignificant differences in weights of the liver, spleen, and right kidney, respectively, of all the experimental rats (both sexes) (*P* > 0.05; Table 6). Likewise, the weights of the right kidneys of male rats were comparable to those of the female rats (*P* > 0.05; Table 6).

### 3.8. Histopathological Examination of Selected Organs

**Figure 2:**
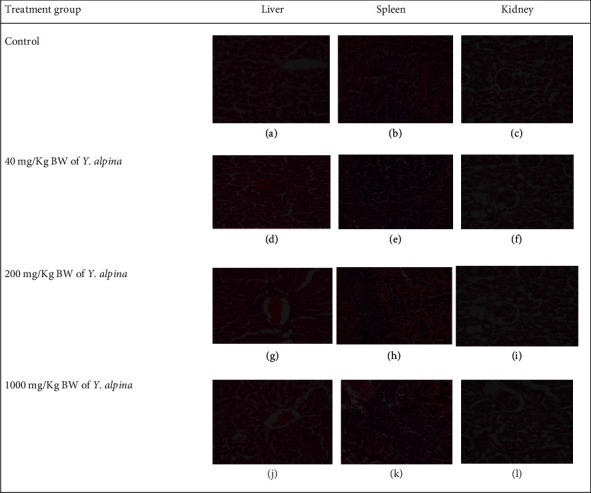
(a–l) Photomicrographs showing histological traits of organs derived from the male rats (haematoxylin-eosin staining; ×400).

**Figure 3:**
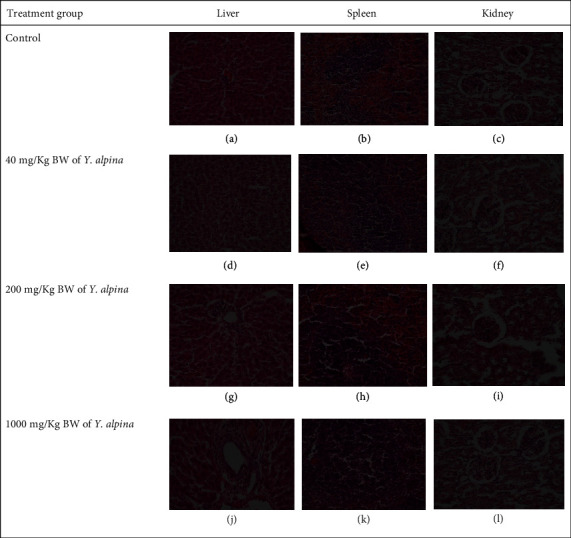
(a–l) Photomicrographs showing histological traits of organs derived from the female rats (haematoxylin-eosin staining; ×400).

Representative photomicrographs of the liver, spleen, and kidney of experimental rats treated with the studied plant extract are presented in Figures 2(a)–2(l) and 3(a)–3(l), respectively. Histological examination of these organs revealed no extract-induced morphological aberrations in all the tissues obtained from experimental rats (both sexes).

## 4. Discussion

Herbalism has been practised since antiquity and forms a critical component of meeting primary healthcare needs in resource-limited regions of the world, especially in Sub-Saharan Africa [1, 7, 21]. Plant-based extracts and products are presumed safe with low or no associated adverse effects when treating various diseases [7]. However, there is scanty empirical evidence to appraise various medicinal plants' toxicity profiles and safety, such as *Y. alpina*, which are used ethnomedicinally as safe remedies for various maladies. There are no specific, conventionally harmonised guidelines for the preparation, packaging, storage, distribution, and dosage in traditional medicine, which raises public health and safety concerns [22]. In addition, there is insufficient pharmacologic data, including herb-herb and herb-synthetic drug interactions, specific indications and contraindications, and associated side effects on various ethnomedicinally important plants like *Y. alpina*, which hamper their appraisal and potential for integration into the conventional healthcare system [9, 23, 24]. Accordingly, we investigated the subacute toxicity effects of the aqueous shoot extract of *Y. alpina* to appraise its safety, owing to its extensive ethnomedicinal applications.

Considering that extracts of *Y. alpina* are taken orally for substantial periods to treat various diseases, we adopted the subacute toxicity study guideline document 407, posited by the OECD [18], to appraise its safety. In this study, experimental animals, preferably the Sprague Dawley rats, are orally administered with the extract daily for 28 days and observed for any signs of toxicity. Besides, this technique provides critical information on extract-induced toxic effects on animal behaviour, physiology, and health marker profiles of clinical biochemistry, haematology, and histopathology for safety characterisation and to guide further research.

This study did not observe any extract-induced behavioural, neurological, or other clinical signs of toxicity in Sprague Dawley rats (male and female) throughout the 28-day experimentation period. Earlier studies show that the absence of clinical signs of toxicity in experimental animals may indicate the safety of the administered agent [25, 26]. Also, the experimental rats' daily water and feed intake remained normal during the study period, depicting the noninterference by the administered extract. Thus, our observations suggest the safety of the aqueous shoot extract of *Y. alpina*, depicting its therapeutic potential.

Body weight is an important anthropometric measure for assessing the toxicity of administered drug agents [27]. Reductions in body weight gain, or lack thereof, are associated with potential exposure to toxic agents, which may cause adverse sequelae. In our study, the body weights of experimental rats treated with the aqueous shoot extract of *Y. alpina* were comparable to those of the control group rats and increased normally during the experimentation period. These findings demonstrate the nontoxicity of the study extract in Sprague Dawley rats, which may signify safety in humans, as previously reported [27, 28].

Determination of the levels of haematological traits offers sensitive and crucial data to assess the degree of toxicity due to exposure to poisonous agents [29]. Clinically, various blood components, commonly referred to as haematologic parameters, including the white blood cells, red blood cells, haematocrit, haemoglobin, mean corpuscular haemoglobin concentration, and platelets, are analysed to determine the presence and extent of toxicity induced by the test agent. The various haematological traits perform critical roles in maintaining normal physiology and health. For instance, the white blood cells and their associated types are responsible for fighting infections and fostering immunity. Low white blood cell counts characterise leukocytopenia, associated with reduced immunity and susceptibility to infections, while sufficiently elevated white blood cell counts indicate leukocytosis and inflammation caused by infections or drug-induced reactions. In our study, the white blood cell counts in Sprague Dawley rats which were subacutely treated with the aqueous shoot extract of *Y. alpina* at all the studied dose levels were normal and comparable to those recorded in the control group rats. These findings suggest that the extract does not induce leukocytopenia and leukocytosis, depicting its potential nontoxicity, thus corroborating previous reports [26, 30].

The red blood cells, haematocrit—the fraction (percentage) of red blood cells in the blood, haemoglobin, and mean corpuscular haemoglobin concentration—are essential markers of blood's oxygen and nutrient carrying capacity in the body [31]. Determination of the levels of these haematological traits is vital in diagnosing anaemia, polycythaemia, and bone marrow capacity to produce blood cells. Previous research shows that exposure to toxic agents causes anaemia, among other blood-related disorders, leading to life-threatening sequelae [25, 26]. Besides, platelets are associated with blood clotting, preventing excessive blood loss, and wound healing following injury. Thrombocytopenia (low platelet count) causes increased blood clotting time and is associated with life-threatening sequelae if not adequately and promptly controlled. Conversely, thrombocytosis (elevated platelet count) is primarily transient and benign; however, it is also linked with haematologic cancer [32]. In our study, all the measured haematologic parameters in experimental rats administered with the aqueous shoot extract of *Y. alpina* were normal and comparable to those of the control group rats, depicting its safety.

Determining biochemical parameters in toxicological investigations offers valuable diagnostic information due to their sensitivity and response to toxicants and clinical symptoms [33]. One of the most crucial functions of the liver involves drug metabolism, biotransformation, and detoxification, making it susceptible to drug-induced toxicity. Besides, the kidney is responsible for the excretion of waste products and drugs from the body, which may lead to its impairment. Thus, measuring liver and kidney function markers is particularly useful in deciphering drug-induced toxicity and appraising the safety of promising drug agents [34].

Urea, creatinine, protein, and albumin levels are important kidney function and health indicators [33, 34]. In acute and chronic renal disease, plasma urea levels are elevated due to impaired clearance and failure of the kidney. Besides, creatinine is an important marker for glomerular filtration efficiency. Reduced creatinine clearance or filtration rate leads to its elevation in plasma, indicating impaired kidney function. Serum protein and albumin levels depend on the amount of water in the blood (volemia) and the concentration of specific proteins. Elevated serum protein and albumin levels are associated with dehydration, which may result from inadequate water intake, excessive loss due to excessive vomiting or diarrhoea, and gastrointestinal irritation by infections or toxicants [28]. In this study, the concentrations of kidney function markers (urea, creatinine, protein, and albumin) in Sprague Dawley rats treated with the aqueous shoot extract of *Y. alpina* were normal compared to those of the control rats. Indeed, the absence of histopathological alterations in the experimental rats' kidney sections indicates the nontoxicity nature of the studied plant extract.

In this study, we also measured alanine aminotransferase (ALT) and aspartate transaminase (AST), which are important markers of liver function, in experimental rats treated with the aqueous shoot extract of *Y. alpina* to determine its effects on liver health and function. Notably, ALT is primarily domiciled in the liver, making it the most sensitive marker of liver damage [35, 36]. Even though AST is found in the liver, kidney, and cardiac and skeletal muscles, determining its levels coupled to ALT is vital in diagnosing cell damage [37]. We observed no significant alterations in ALT and AST enzyme levels in experimental rats treated with the aqueous shoot extract of *Y. alpina,* and the measured levels were comparable to those of control group rats. These observations indicate the nontoxicity nature of the studied plant extract, which was further substantiated by the lack of histopathological aberration in liver sections of the experimental rats.

The liver and kidney are prone to drug- or toxicant-induced cell damage due to their fundamental roles in metabolism and excretion [35, 37]. The spleen is the principal organ of the lymphatic system, which plays an essential role in producing and manipulating crucial immunity elements. Intact drugs, exogenous chemical toxicants, or their metabolites might cause toxicity to these organs (liver, kidney, and spleen) depending on their dose and period of exposure, resulting in characteristic histopathological changes [37]. Damage to these organs may result in deleterious consequences, including death.

In this study, gross necroscopy examination of various organs, including the liver, spleen, and kidneys, in experimental rats treated with the aqueous shoot extract of *Y. alpina* revealed no textural, morphological, or any other visible pathological or toxicological signs. In addition, no pathologic changes were observed in the experimental rats' liver, kidney, and spleen sections. These findings depict the safety of the studied plant extract, at least in Sprague Dawley rats, in line with previous studies [24, 26, 33, 38] and are further supported by the biochemical, haematological, and physical examination evidence obtained in our study. Moreover, the safety of the studied plant extract may be attributable to the low concentration or absence of some amalgams, which have been demonstrated previously to cause toxicity [39, 40].

Considering the longstanding utilisation of *Y. alpina* shoots to treat various diseases, including diarrhoea, inflammation, and infections, in traditional medicine [12–15], their toxicity profile and safety have been unknown. Accordingly, our study provides empirical evidence to validate the safety of *Y. alpina,* spur further research, and valorise it as a potential source of safe therapeutic remedies. Furthermore, empirical investigation and appraisal of its safety are imperative considering the widespread acceptability of medicinal plants and their usage by over 80% of the world population to treat various diseases.

## 5. Conclusions and Recommendations

This study demonstrates that the aqueous shoot extract of *Yushania alpina* does not cause subacute toxicity in Sprague Dawley rats, as revealed by the wellness, haematological, biochemical, necroscopy, and histopathological findings. Therefore, the studied plant extract may be a safe source or remedy for various diseases it is claimed to treat in traditional medicine. Further toxicological investigations using other animal models and in a clinical setup are encouraged to demystify its safety exhaustively. Moreover, empirical investigations are recommended to validate the pharmacologic efficacy and mode(s) of action of *Y. alpina* in various disease states where it is used as a remedy.

## Figures and Tables

**Table 1:** Average daily feed intake.

Treatment	Average daily feed intake (g)	
	Male	Female
Control	114.62 ± 3.54^a^_a_	90.07 ± 2.00^a^_b_
40 mg/Kg BW of *Y. alpina*	106.07 ± 2.42^ab^_a_	80.20 ± 1.36^b^_b_
200 mg/Kg BW of *Y. alpina*	105.89 ± 2.89^ab^_a_	86.67 ± 1.05^ab^_b_
1000 mg/Kg BW of *Y. alpina*	101.76 ± 2.58^b^_a_	86.00 ± 2.73^ab^_b_

Values are presented as $\bar{x}\pm SEM$ of replicate measurements (28 days). Means with similar superscript alphabets within the same column are not significantly different (*P* > 0.05; One-Way ANOVA with Tukey's *Post hoc*); means with different subscript alphabets within the same row are significantly different (*P* > 0.05; unpaired Student's *t*-test); control group rats received distilled water (10 ml/Kg BW; *p.o*).

**Table 2:** Average daily water intake.

Treatment	Average daily water intake (ml)	
	Male	Female
Control	143.21 ± 6.83^a^_a_	90.07 ± 2.00^a^_b_
40 mg/Kg BW of *Y. alpina*	137.86 ± 7.01^a^_a_	80.20 ± 1.36^b^_b_
200 mg/Kg BW of *Y. alpina*	146.07 ± 7.52^a^_a_	86.67 ± 1.05^ab^_b_
1000 mg/Kg BW of *Y. alpina*	128.75 ± 8.73^a^_a_	86.00 ± 2.73^ab^_b_

Values are presented as $\bar{x}\pm \text{SEM}$ of replicate measurements (28 days). Means with similar superscript alphabets within the same column are not significantly different (*P* > 0.05; One-Way ANOVA with Tukey's *Post hoc*); means with different subscript alphabets within the same row are significantly different (*P* < 0.05; unpaired Student's *t*-test); control group rats received 10 ml/Kg BW of distilled water orally.

**Table 3:** Weekly body weights.

Treatment group	Body weight (g)				
	Week 0	Week 1	Week 2	Week 3	Week 4
Male rats					
Control	183.81 ± 8.47^a^_c_	215.60 ± 10.90^a^_bc_	293.30 ± 13.80^a^_ab_	261.10 ± 12.50^a^_ab_	272.20 ± 11.30^a^_a_
40 mg/Kg BW of *Y. alpina*	191.16 ± 9.31^a^_b_	230.30 ± 16.90^a^_ab_	241.20 ± 17.80^a^_ab_	259.70 ± 15.50^a^_a_	274.90 ± 14.00^a^_a_
200 mg/Kg BW of *Y. alpina*	193.07 ± 5.71^a^_d_	226.42 ± 5.49^a^_c_	241.55 ± 4.69^a^_bc_	258.21 ± 4.87^a^_ab_	273.39 ± 3.64^a^_a_
1000 mg/Kg BW of *Y. alpina*	188.32 ± 4.54^a^_c_	224.21 ± 7.06^a^_bc_	244.68 ± 9.98^a^_ab_	261.00 ± 10.70^a^_ab_	277.73 ± 9.90^a^_a_

Female rats					
Control	177.31 ± 6.20^a^_c_	204.90 ± 8.10^a^_b_	229.30 ± 6.65^a^_ab_	244.11 ± 5.08^a^_a_	255.41 ± 4.85^a^_a_
40 mg/Kg BW of *Y. alpina*	182.16 ± 9.66^a^_b_	206.10 ± 10.40^a^_ab_	228.90 ± 13.10^a^_ab_	243.70 ± 13.50^a^_a_	253.40 ± 12.30^a^_a_
200 mg/Kg BW of *Y. alpina*	175.48 ± 8.03^a^_b_	209.86 ± 9.82^a^_ab_	225.60 ± 11.20^a^_a_	238.20 ± 11.20^a^_a_	250.00 ± 11.40^a^_a_
1000 mg/Kg BW of *Y. alpina*	171.78 ± 3.92^a^_d_	203.15 ± 5.75^a^_c_	225.50 ± 5.79^a^_bc_	243.36 ± 6.71^a^_ab_	254.05 ± 6.01^a^_a_

Values are expressed as $\bar{x}\pm \text{SEM}$ of replicate measurements. Means with similar superscript alphabets within the same column and those with the same subscript alphabets within the same row are not significantly different (*P* > 0.05, One-Way ANOVA with Tukey's *Post hoc*); control group rats received 10 ml/Kg BW of distilled water orally.

**Table 4:** Haematological traits.

Treatment group	Haematologic traits					PLT (10^9^/L)
	WBC (10^9^/L)	RBC (10^12^/L)	Hb (g/dL)	MCHC (g/dL)	HCT (%)	
Male rats						
Control	11.34 ± 2.38^a^	11.44 ± 1.92^a^	10.98 ± 1.74^a^	16.10 ± 0.71^a^	70.30 ± 12.70^a^	970.00 ± 351.00^ab^
40 mg/Kg BW of *Y. alpina*	13.75 ± 1.01^a^	12.60 ± 0.10^a^	12.10 ± 0.26^a^	14.92 ± 0.14^a^	81.78 ± 1.85^a^	843.00 ± 126.00^b^
200 mg/Kg BW of *Y. alpina*	12.89 ± 1.40^a^	11.69 ± 0.75^a^	11.54 ± 0.52^a^	15.78 ± 0.45^a^	74.38 ± 4.49^a^	1068.00 ± 102.00^ab^
1000 mg/Kg BW of *Y. alpina*	11.24 ± 1.51^a^	12.51 ± 0.75^a^	11.30 ± 0.43^a^	14.04 ± 0.67^a^	81.68 ± 6.42^a^	1623.00 ± 127.00^a^

Female rats						
Control	11.20 ± 2.24^a^	10.80 ± 0.84^a^	12.82 ± 0.99^a^	19.66 ± 2.92^a^	67.58 ± 4.33^a^	905.00 ± 82.00^ab^
40 mg/Kg BW of *Y. alpina*	12.63 ± 1.91^a^	13.89 ± 2.78^a^	11.66 ± 0.72^a^	13.60 ± 1.72^a^	97.20 ± 23.20^a^	1279.00 ± 99.10^ab^
200 mg/Kg BW of *Y. alpina*	13.17 ± 3.32^a^	12.46 ± 1.31^a^	12.60 ± 0.83^a^	16.62 ± 3.23^a^	81.32 ± 7.39^a^	1123.00 ± 81.70^ab^
1000 mg/Kg BW of *Y. alpina*	16.42 ± 4.31^a^	10.61 ± 1.31^a^	13.74 ± 1.24^a^	20.28 ± 3.77^a^	72.90 ± 7.25^a^	790.00 ± 103.00^b^

Values are presented as $\bar{x}\pm SEM$ of replicate experiments. Means with similar superscript alphabets within the same column are not significantly different (*P* > 0.05, One-Way ANOVA with Tukey's *Post hoc*); control group rats received 10 ml/Kg BW of distilled water orally. WBC: white blood cells; RBC: red blood cells; Hb: haemoglobin; MCHC: mean corpuscular haemoglobin concentration; HCT: haematocrit; PLT: platelets.

**Table 5:** Biochemical parameters.

Treatment group	Serum biochemical parameters					P (g/L)	A (g/dL)
	U (mmol/L)	C (*μ*mol/L)	ALP (U/L)	ALT (U/L)	AST (U/L)		
Male rats							
Control	10.40 ± 0.44^a^	61.53 ± 1.62^ab^	152.40 ± 12.50^ab^	80.20 ± 7.81^a^	270.40 ± 15.10^a^	72.35 ± 1.34^ab^	3.67 ± 0.08^a^
40 mg/Kg BW of *Y. alpina*	10.58 ± 0.84^ab^	63.45 ± 1.85^ab^	208.20 ± 30.00^ab^	64.20 ± 2.58^a^	242.00 ± 11.20^a^	67.16 ± 1.72^b^	3.45 ± 0.21^a^
200 mg/Kg BW of *Y. alpina*	10.51 ± 0.72^ab^	58.35 ± 1.02^b^	171.00 ± 16.50^ab^	63.80 ± 5.28^a^	311.00 ± 34.90^a^	68.96 ± 1.63^ab^	3.47 ± 0.09^a^
1000 mg/Kg BW of *Y. alpina*	12.21 ± 0.49^ab^	62.94 ± 1.75^ab^	245.60 ± 43.90^a^	73.20 ± 13.80^a^	288.40 ± 37.90^a^	68.63 ± 3.20^ab^	3.34 ± 0.15^a^

Female rats							
Control	7.72 ± 0.60^b^	61.53 ± 3.04^ab^	81.20 ± 19.10^b^	72.80 ± 9.21^a^	213.60 ± 9.93^a^	76.79 ± 1.71^ab^	3.85 ± 0.13^a^
40 mg/Kg BW of *Y. alpina*	8.96 ± 0.56^b^	70.52 ± 3.64^a^	112.80 ± 43.90^b^	55.20 ± 6.50^a^	234.60 ± 36.90^a^	77.73 ± 4.36^a^	3.76 ± 0.18^a^
200 mg/Kg BW of *Y. alpina*	8.35 ± 0.71^b^	61.68 ± 1.41^ab^	151.80 ± 19.90^ab^	63.00 ± 5.30^a^	263.40 ± 17.40^a^	76.21 ± 0.17^ab^	3.64 ± 0.12^a^
1000 mg/Kg BW of *Y. alpina*	8.35 ± 0.71^b^	61.68 ± 1.41^ab^	151.80 ± 19.90^ab^	63.00 ± 5.30^a^	263.40 ± 17.40^a^	76.21 ± 0.17^ab^	3.64 ± 0.12^a^

Values are presented as $\bar{x}\pm \text{SEM}$ of replicate experiments. Means with similar superscript alphabets within the same column are not significantly different (*P* > 0.05, One-Way ANOVA with Tukey's *Post hoc*); control group rats received 10 ml/Kg BW of distilled water orally. U: urea; C: creatinine; ALP: alkaline phosphate; ALT: alanine aminotransferase; AST: aspartate transaminase; P: total protein; A: albumin.

**Table 6:** Selected organ weights.

Treatment group	Organ weights			
	Liver	Spleen	Kidney (L)	Kidney (R)
Male rats				
Control	8.53 ± 0.49^a^	1.08 ± 0.09^a^	0.91 ± 0.09^a^	0.90 ± 0.08^a^
40 mg/Kg BW of *Y. alpina*	8.54 ± 0.59^a^	0.97 ± 0.08^a^	0.89 ± 0.06^ab^	0.89 ± 0.07^a^
200 mg/Kg BW of *Y. alpina*	8.06 ± 0.68^a^	1.16 ± 0.11^a^	0.89 ± 0.05^ab^	0.84 ± 0.04^a^
1000 mg/Kg BW of *Y. alpina*	7.99 ± 0.55^a^	1.20 ± 0.10^a^	0.85 ± 0.05^ab^	0.88 ± 0.04^a^

Female rats				
Control	8.13 ± 0.28^a^	1.02 ± 0.08^a^	0.88 ± 0.02^ab^	0.88 ± 0.02^a^
40 mg/Kg BW of *Y. alpina*	7.25 ± 0.51^a^	0.85 ± 0.11^a^	0.74 ± 0.05^ab^	0.74 ± 0.08^a^
200 mg/Kg BW of *Y. alpina*	6.25 ± 0.57^a^	0.95 ± 0.06^a^	0.67 ± 0.03^b^	0.72 ± 0.03^a^
1000 mg/Kg BW of *Y. alpina*	7.12 ± 0.59^a^	0.90 ± 0.04^a^	0.73 ± 0.02^ab^	0.68 ± 0.02^a^

Values are expressed as $\bar{x}\pm SEM$ of replicate experiments. Means with similar superscript alphabets within the same column are not significantly different (*P* > 0.05, One-Way ANOVA with Tukey's *Post hoc*); control group rats received distilled water (10 ml/Kg BW; *p.o*).

## Data Availability

All the data used to support the ﬁndings of this study are included in the article. The authors may offer any additional information upon reasonable request.
